# How prognostic information influences care planning in adult intensive care units: protocol for a realist review

**DOI:** 10.1136/bmjopen-2026-117449

**Published:** 2026-06-29

**Authors:** Arunangshu Ghoshal, Ashmitha Prasad, Aparna Nanda, Naveen Salins

**Affiliations:** 1 Department of Palliative Medicine and Supportive Care, Kasturba Medical College, Manipal Academy of Higher Education, Manipal, India - 576104

**Keywords:** Adult intensive & critical care, Adult palliative care, HEALTH SERVICES ADMINISTRATION & MANAGEMENT

## Abstract

**Abstract:**

**Introduction:**

Prognostic information plays a key role in decision-making in adult intensive care units (ICUs), but its influence on advance care planning, treatment limitation and palliative-care integration depends on organisational, legal, cultural and relational factors. This review focuses on how prognostic information is used in decision-making processes. This realist review will explore how, why, for whom and in which ICU contexts prognostic information impacts care planning.

**Methods and analysis:**

Following Realist and Meta-narrative Evidence Syntheses (RAMESES) publication standards for realist syntheses, we will iteratively develop an initial programme theory (IPT) and refine context-mechanism-outcome (CMO) configurations through staged database and grey literature searches, selecting sources based on relevance and rigour, performing theory-driven extraction and applying realist synthesis strategies (juxtaposition, reconciliation, adjudication and consolidation). Stakeholders, including ICU and palliative care clinicians, caregiver representatives and ethics/policy advisors, will contribute to shaping IPT development, CMO refinement and interpretation. Reporting will also adhere to Preferred Reporting Items for Systematic Review and Meta-Analysis Protocols (PRISMA-P) guidelines; the completed checklist is provided as Supplementary File S1.

**Ethics and dissemination:**

Ethical approval is not required (secondary analysis).

**Dissemination:**

Findings will be disseminated through peer-reviewed publications, conference presentations and stakeholder-targeted briefs for clinicians, policymakers and professional bodies.

**Registration:**

Open Science Framework (OSF: https://osf.io/6qu8x).

**STRENGTHS AND LIMITATIONS OF THIS STUDY**

- This realist review will use an explicit, theory-driven methodology (Realist and Meta-narrative Evidence Syntheses (RAMESES) standards) with iterative development and refinement of context-mechanism-outcome configurations.
- The review will incorporate systematic, staged searching across multiple databases and grey literature sources, with search strategies peer-reviewed using PRESS guidelines.
- Dual independent screening, theory-informed data extraction and transparent documentation of appraisal decisions will enhance methodological rigour.
- The review will include stakeholder engagement at multiple stages to refine programme theory and interpret findings.
- Limitations include restriction to English-language sources and potential variability in the reporting of mechanisms across included studies.

## Introduction

 Prognostication—the process of estimating a patient’s likely clinical course and outcome—has traditionally been vital to decision-making in intensive care units (ICUs). It also plays a crucial role in advance care planning (ACP) by setting expectations, guiding goals-of-care discussions and impacting decisions about limiting treatment.^1^ In patients who are critically ill, particularly those with advanced or life-limiting conditions, prognostic information shapes decisions regarding treatment escalation or limitation, the appropriateness of life-sustaining interventions and the timing and nature of palliative care (PC) involvement.^2^,^4^ These decisions unfold in high-stakes environments where clinicians, patients and families navigate complex emotional, ethical and informational terrain, balancing the imperative to prolong life against the need to alleviate suffering.^5^ Despite its central role, ICU prognostication remains characterised by uncertainty, variability and ongoing debate.^6^

ICU prognostication encompasses three interrelated components. First, severity-of-illness models such as APACHE II (Acute Physiology and Chronic Health Evaluation) and SAPS II (Simplified Acute Physiology Score) estimate mortality risk at the population level and are primarily used for benchmarking and quality assessment rather than individual prediction.^7 8^ Second, organ dysfunction scores such as Sequential Organ Failure Assessment (SOFA) track evolving physiological derangements and provide insight into clinical trajectories.^9^ Third, clinicians rely on experiential judgement, integrating comorbidities, frailty, premorbid function and patient values into interpretive assessments.^10^ While these elements inform decision-making, none independently determine care pathways. Instead, prognostic information is interpreted through processes such as serious illness communication, shared decision-making and ACP, which mediate how estimates are understood and acted on.^5 11 12^ This is significant given the scale and consequences of ICU decision-making.

ICU mortality globally ranges from approximately 10%–30%,^13^ with higher rates in low- and middle-income countries.^14^,^16^ A substantial proportion of deaths follow decisions to withhold or withdraw life-sustaining treatments, often accounting for 50%–90% of ICU deaths.^17^ At the same time, ACP remains poorly integrated into ICU populations, and non-beneficial treatments are common.^118^,^20^ Although international guidelines emphasise structured end-of-life (EOL) decision-making, family-centred communication and early PC involvement,^121^,^25^ the pathways through which prognostic information influences such processes vary widely.

Contextual factors play a critical role in shaping these pathways. Prognostic information may activate mechanisms such as trust, shared moral reasoning or acceptance in some settings, while in others it may trigger fear of blame, conflict avoidance or moral distress.^2 11 26 27^ In jurisdictions with restrictive legal frameworks, clinicians may hesitate to communicate a poor prognosis explicitly due to concerns about litigation or institutional scrutiny.^28^ Cultural norms may further shape how families engage in discussions about dying, with some preferring indirect communication and others valuing transparency.^29^,^31^ These variations challenge linear assumptions that improved prognostic accuracy will automatically lead to better decision-making. Instead, they highlight the need to understand how context shapes the mechanisms through which prognostic information exerts its effects.

These issues are particularly salient in India, where ICU practice is heterogeneous and influenced by evolving legal frameworks, professional guidance and resource variability.^19 20^ Differences in institutional capacity, clinician training, family expectations and medico-legal environments complicate the translation of prognostic estimation into clinical action.^28 32 33^ Here, we distinguish between critical illness with uncertain reversibility, advanced life-limiting illness and EOL states, where death is expected, recognising that prognostic information serves different functions across these contexts.

A realist review is well-suited to address these complexities. Realist reviews are theory-driven approaches that seek to explain how and why interventions work (or fail) in specific contexts.^34^ Rather than asking whether an intervention works, realist methodology seeks to explain how, why, for whom and under what circumstances it works. It conceptualises interventions as theories-in-action and examines how context interacts with underlying mechanisms to produce outcomes.^35^,^37^ In ICU prognostication, the implicit assumption is that accurate prognostic information leads to timely, appropriate decisions regarding treatment limitation and ACP. However, this holds only under specific conditions—such as when information is perceived as credible, communication is effective and organisational cultures support patient-centred care. In other contexts, the same information may lead to delayed decision-making or continuation of non-beneficial treatment.

In this review, we distinguish between (1) acute critical illness with uncertain reversibility, (2) advanced life-limiting illness and (3) EOL states where death is expected. Prognostic tools and clinical judgement serve different purposes across these contexts. For example, severity scoring systems (eg, APACHE) estimate population-level mortality risk, whereas organ dysfunction scores (eg, SOFA) track clinical trajectories. This review focuses not on the predictive performance of such tools, but on how prognostic information—whether model-based or experiential—is interpreted and used within care-planning processes such as ACP and treatment limitation.

## Aim and objectives

This review aims to synthesise how prognostic information is generated, interpreted and communicated in adult ICUs and how it varies across clinical and organisational contexts. It will identify mechanisms through which prognostic information influences care-planning decisions and develop context-mechanism-outcome (CMO) configurations to explain variation in outcomes. The review will also generate theory-informed, context-sensitive recommendations for practice, education and governance.

## Methods and analysis

### Realist methodological approach

We will conduct a realist review in line with Realist and Meta-narrative Evidence Syntheses (RAMESES) publication standards for realist syntheses. Reporting will also adhere to Preferred Reporting Items for Systematic Review and Meta-Analysis Protocols (PRISMA-P) 2015; a completed PRISMA-P checklist is provided as an online supplemental file S1. All decisional steps will be logged on Open Science Framework (OSF) to ensure auditability. We anticipate inclusion of approximately 40–80 studies, with sampling guided by theoretical saturation rather than exhaustiveness.

### Initial programme theory

The initial programme theory (IPT) was developed through preliminary scoping of the literature on ICU prognostication and EOL care, review of international guidelines and discussions within the multidisciplinary research team.^1^ No formal stakeholder consultation informed the initial theory at this stage. The IPT proposes that prognostic information influences ICU care planning when it is perceived as credible, ethically legitimate and shared among clinicians and families.

**Figure 1:**
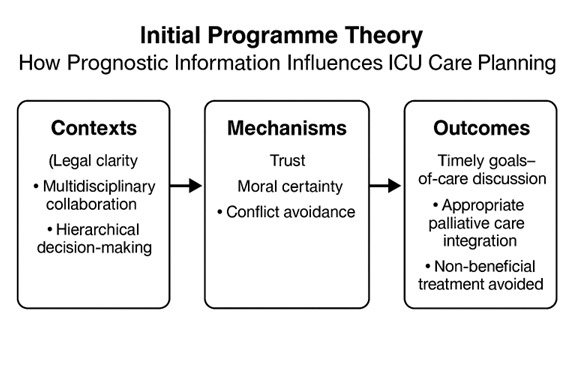
Initial programme theory: hypothesised context-mechanism-outcome configurations explaining how prognostic information influences care planning decisions in intensive care unit (ICU) settings. Note: contexts activate mechanisms through which prognostic information influences outcomes relevant to care planning (advance care planning, treatment limitation and palliative integration).

Candidate CMOs are summarised in figure 1 and include:

- Where policies/legal clarity supports treatment limitation, prognostic information is perceived as legitimate → mechanisms: clinician confidence and reduced fear of blame → outcomes: timely goals-of-care and earlier palliative involvement.
- Where structured family meetings are routine, prognostic information fosters shared understanding → mechanisms: trust and moral alignment → outcomes: consensus and reduced conflict.
- Where hierarchy is rigid, and litigation fears are high, identical prognostic cues trigger defensive reasoning → mechanisms: conflict avoidance and moral distress → outcomes: delayed limitation and non-beneficial treatments.

### Search strategy

We will undertake staged searching across MEDLINE (Ovid), Embase (Ovid), CINAHL (EBSCOhost), PsycINFO (Ovid), Scopus (Elsevier), Web of Science (Clarivate) and the Cochrane Library (Wiley), complemented by purposive grey literature searches (professional guidance, legal/regulatory materials, institutional policies and theses). Search results will be imported into reference management software (eg, Zotero), where automatic de-duplication will be performed. Additional manual de-duplication will be conducted by reviewers during the screening process to ensure accuracy. A benchmark set of key sources will validate sensitivity, a PRESS-informed peer review will be performed and responsive searching will continue as CMOs evolve. All search strings, dates and decisions will be publicly archived. The literature search will be conducted between June 2026 and August 2026. At the time of submission, the review is at the protocol stage, and searches have not yet been completed.

### Eligibility and study selection

Inclusion: adult ICU settings; concepts covering prognostic practices (models, scores and clinical judgement), communication, decision-making and ACP/EOL processes; and designs spanning qualitative, quantitative, mixed methods, reviews, theory and policy. The review will be restricted to English-language publications due to resource constraints and limited access to translation services; however, this may limit the inclusion of relevant studies from non-English-speaking contexts. Exclusion: paediatric/neonatal ICUs; model development without decision relevance. Two reviewers (AG and AP) will independently screen records; disagreements will be resolved through discussion or consultation with a third reviewer (NS); reasons for exclusion will be logged.

### Appraising rigour and relevance

Following realist logic, relevance denotes the capacity to inform, test or refine CMOs; rigour is the trustworthiness of the data for the specific inference. We will draw pragmatically on design-specific tools (eg, CASP) to inform—but not score—judgements. We will transparently document how rigour judgements influenced theory development.

### Data extraction

We will extract the following: settings, participants, prognostic practices, contexts (organisational, legal/policy and cultural), candidate mechanisms (reasoning/responses) and outcomes (care planning, treatment limitation, palliative-care integration and family/system outcomes). A CMO template will be used to capture explanatory propositions and quotes.

### Synthesis and theory refinement

Data related to contexts, mechanisms and outcomes will be coded both inductively and deductively. The initial coding will follow the IPT framework, with new themes added as they arise. CMO configurations will be created by connecting contextual factors, mechanisms and outcomes observed across different studies. We will iteratively develop these configurations through methods such as juxtaposition (comparing sources to generate insights), reconciliation (explaining differences in outcomes via contextual factors), adjudication (assessing the reliability and richness of data) and consolidation (creating multi-level explanations). Stakeholder input will be used to evaluate competing theories and identify boundary conditions. In line with established realist review methodology, the final stage will involve developing an explanatory narrative and formulating context-sensitive recommendations derived from the refined programme theory.

### Stakeholder engagement and governance

We will recruit ~15–20 stakeholders: 6–8 ICU clinicians (consultants, nurses and trainees), 3–4 palliative-care clinicians, 3–5 family caregiver representatives and two ethics/policy advisors. Engagement: (T1) IPT elicitation (months 0–2); (T2) CMO refinement (months 3–6) and (T3) interpretation/recommendations (months 5–7). Recruitment via institutional departments and networks and bilingual facilitation and anonymous polling will mitigate hierarchy effects; consent/honoraria per institutional policy. Here, T1 and T2 refer to specific, time-bound phases of the research.

### Patient and public involvement

Patient and public involvement was not feasible during early protocol development due to time and resource constraints. However, stakeholders, including caregiver representatives, will be actively involved in subsequent stages of the review, including programme theory refinement, interpretation and development of recommendations.

The final stage of the review will involve developing a narrative synthesis and formulating context-sensitive recommendations based on a refined programme theory.

## Ethics and dissemination

This review will use published/grey literature and does not require ethical approval. Stakeholder activities will be reviewed in accordance with institutional procedures. Informed consent will be obtained from participants and no identifiable personal data will be reported.

Dissemination will include peer-reviewed publications, conference presentations and targeted outputs (eg, policy briefs and educational materials) tailored to clinicians, professional societies and health system stakeholders.

### Transparency, data availability and protocol amendments

We will make search strategies, screening decisions, extraction templates and analytic memos available as supplementary materials or via OSF. Any substantive protocol amendments will be documented with dates, rationale and implications for synthesis.

## Supplementary material

10.1136/bmjopen-2026-117449 online supplemental file 1
