# Supplementary material for: How prognostic information influences care planning in adult intensive care units: protocol for a realist review
Source: BMJ Open. 2026 Jun 29;16(6):e117449. doi: 10.1136/bmjopen-2026-117449 (PMC13343029; doi:10.1136/bmjopen-2026-117449)
Supplement: online supplemental file 1 [file bmjopen-16-6-s001.docx]

# Supplementary File S1. PRISMA-P 2015 Checklist

| Item | Topic | Checklist item | Location in manuscript |
| --- | --- | --- | --- |
| 1a | Identification | Title identifies the report as a protocol of a realist review | Page 1, lines 1-2 |
| 1b | Update | If an update, identify as such | Not applicable |
| 2 | Registration | Register the protocol | Page 2, line 20 |
| 3a | Authors | Contact information | Page 1, lines 4-22 |
| 3b | Contributions | Author contributions | Page 13; lines 1-7 |
| 4 | Amendments | Plan for documenting amendments | Not applicable |
| 5a | Support | Sources of financial support | Page 12; lines 1-7 |
| 5b | Sponsor | Sponsor/funder role | Page 12; lines 6-7 |
| 5c | Roles | Sponsor/funder roles and competing interests | Page 12; lines 8-9 |
| 6 | Rationale | Describe rationale for the review | Page 5; lines 9-18 |
| 7 | Objectives | Provide explicit PICO/STaR objectives | Page 7; lines 5-10 |
| 8 | Eligibility criteria | Study characteristics and report characteristics | Page 9; lines 1-9 |
| 9 | Information sources | All intended sources | Page 8; lines 14-24 |
| 10 | Search strategy | Draft search strategy for at least one database | Not applicable |
| 11a | Study records – data management | How records/data will be managed | Page 9; lines 16-19 |
| 11b | Study records – selection | Selection process | Page 9; lines 1-9 |
| 11c | Study records – data collection | Planned data collection process | Page 9; lines 16-19 |
| 12 | Risk of bias in individual studies | Anticipated assessment | Page 9; lines 11-14 |
| 13 | Data synthesis | Planned synthesis methods | Page 9; lines 21-26, Page 10; lines 1-5 |
| 14 | Meta-bias(es) | Assessment of reporting biases | Not applicable |
| 15a | Confidence in cumulative evidence | Approach to summary confidence | Not applicable |
| 16 | PPI | Patient and public involvement statement | Page 10; lines 13-19 |
