# Supplementary material for: How prognostic information influences care planning in adult intensive care units: protocol for a realist review
Source: BMJ Open. 2026 Jun 29;16(6):e117449. doi: 10.1136/bmjopen-2026-117449 (PMC13343029; doi:10.1136/bmjopen-2026-117449)
Supplement: Supplementary file 2 [file bmjopen-2026-117449.reviewer_comments.pdf]

PEER REVIEW HISTORY

BMJ Open publishes all reviews undertaken for accepted manuscripts. Reviewers are asked to complete a checklist review form and are provided with free text boxes to elaborate on their assessment. These free text comments are reproduced below.

ARTICLE DETAILS

Title (Provisional)

Protocol for a Realist Review: How Prognostic Information Influences Care Planning in Adult Intensive Care Units

Authors

Ghoshal, Arunangshu; Prasad, Ashmitha; Nanda, Aparna; Salins, Naveen

---

VERSION 1 - REVIEW

**Reviewer**	**1**
**Name**	**Manning, Joseph**
**Affiliation**	**University of Leicester, School of Healthcare**
**Date**	**12-Mar-2026**
**COI**	**None**

Thank you for the opportunity to review this manuscript which addresses a potentially important topic. However, in its current form, the protocol lacks sufficient conceptual clarity, methodological detail, and coherence to support publication. The paper would require substantial redevelopment before it could be considered further. My main concerns are set out below.

1. The background and introduction require substantial revision for clarity and consistency.

The introduction is currently unclear in both conceptual and practical terms. The manuscript uses terms such as *terminal illness*, *advanced care*, *palliative care*, and *death and dying* as though they are equivalent, but they are not. These distinctions matter because prognostic tools may serve different purposes depending on the clinical context. For example, APACHE is primarily used to estimate mortality risk in critically ill patients, whereas SOFA is used to assess organ dysfunction and track clinical deterioration. The manuscript does not explain how such tools relate to the stated focus on advance care planning, nor does it identify which tools are relevant in which settings. This creates confusion about the review question and its intended contribution. Moreover, literature pertaining to advanced care planning, end of life care, and death in the ICU context is omitted. The background would also benefit from epidemiological context. At present, there are no data presented to

establish the scale of life-limiting illness, end-of-life care needs, or decision-making challenges in ICU. Without this, the rationale for the review remains weak.

2. The presentation of the realist methodology is insufficiently developed. Some aspects of the methods appear in the background rather than being clearly and systematically described in the methods section. As this is a methods paper, the protocol should provide a more robust and transparent account of the realist approach being used, including how the review will proceed and how decisions will be made at each stage which should be supported by relevant references.

3. There is no clear rationale for the absence of patients, service users, or other stakeholders in protocol development. For a review focused on care planning, prognostication, and decision-making in serious illness, it is notable that no clear justification is provided for the lack of patient, family, public, or wider stakeholder involvement to date. Even if such involvement was not feasible, this should be explained and discussed. At present, this omission weakens the relevance and credibility of the protocol.

4. The manuscript refers to an initial programme theory, but it is not clear how this theory was developed. The reader needs a more explicit account of the sources that informed it, such as preliminary literature scoping, consultation with stakeholders, or theoretical frameworks. In its current form, the programme theory is too vague to guide the review in a meaningful way. This section would benefit from fuller explanation and, ideally, presentation in a diagram or figure to improve transparency.

5. The aims and objectives do not clearly follow from the background. At present, the objectives appear insufficiently anchored in the literature and problem definition provided earlier in the manuscript. This makes the study rationale difficult to follow.

6. It is unclear why the sixth stage of the realist approach, namely developing a narrative and making recommendations, has not been included. If the authors are using a recognised staged realist process, any omissions need to be justified. As written, the description of the review process feels incomplete.

7. The manuscript does not provide enough methodological detail for a protocol paper. Because this is presented as a methods paper, I would expect much more detail on the planned procedures, (appraisal, synthesis, and theory refinement) proposed numbers of stakeholders, how they will be accessed etc and supporting evidence cited to articulate why approach selected . At present, the methods remain too general and do not provide enough evidence of rigour.

8. The manuscript refers to PRISMA-P, but the checklist is not provided as a supplementary file. For completeness and transparency of reporting, this should be included.

9. The manuscript is difficult to read and follow. The paper would benefit from substantial improvement in writing style and structure. The current version contains limited narrative development, frequent single-sentence paragraphs, and list-like presentation throughout.

This disrupts the flow of the argument and makes the manuscript hard to read. The prose needs to be revised to improve coherence, readability, and scholarly presentation.

VERSION 1 - AUTHOR RESPONSE

Reviewer 1

We thank the reviewer for their thoughtful and detailed critique. We have undertaken substantial revisions to address all concerns.

Comment 1: Conceptual clarity and inconsistent terminology

Response:
We have substantially revised the Introduction to clarify key conceptual distinctions between acute critical illness, advanced life-limiting illness, and end-of-life states. We now explicitly describe how different prognostic tools (e.g., APACHE, SOFA, clinical judgement) function across these contexts and clarify that the review focuses on the *use and interpretation* of prognostic information rather than predictive performance. We have also strengthened the epidemiological context to better establish the scale and relevance of the problem.

Comment 2: Insufficient development of realist methodology

Response:
We have reorganized and expanded the Methods section by introducing a dedicated subsection titled "Realist methodological approach." This section now clearly outlines the theoretical basis of realist review, including context–mechanism–outcome (CMO) logic, iterative theory refinement, and adherence to RAMESES standards. We have also clarified how data will be coded and how CMO configurations will be developed during synthesis.

Comment 3: Lack of patient/public involvement justification

Response:
We have added a clear justification explaining that patient and public involvement was not feasible during early protocol development due to resource constraints. However, we now explicitly describe planned stakeholder engagement, including caregiver representatives, in subsequent stages of theory refinement and interpretation.

Comment 4: Unclear development of initial programme theory

Response:
We have clarified that the initial programme theory was developed through preliminary literature scoping, review of international guidance, and multidisciplinary team discussions. We have also strengthened the description of the IPT and improved the accompanying figure to enhance transparency.

Comment 5: Aims and objectives not clearly linked to background

Response:
We have revised the Introduction and refined the Aim and Objectives section to ensure a clearer alignment with the problem definition and conceptual framework presented earlier in the manuscript.

Comment 6: Missing stage of realist review (narrative and recommendations)

Response:
We have explicitly incorporated the final stage of realist review, including development of an explanatory narrative and formulation of context-sensitive recommendations derived from the refined programme theory.

Comment 7: Insufficient methodological detail

Response:
We have expanded methodological detail across sections, including searching strategy, study selection, appraisal, data extraction, and synthesis. We now provide clearer descriptions of analytic processes, including how CMO configurations will be constructed and refined, and have included additional detail on stakeholder engagement.

Comment 8: PRISMA-P checklist not provided

Response:
The completed PRISMA-P checklist has now been included as Supplementary File S1, as required.

Comment 9: Writing style and structure

Response:
We have revised the manuscript extensively to improve clarity, coherence, and readability. This includes restructuring paragraphs, reducing list-like presentation, improving narrative flow, and ensuring consistency in terminology and academic tone.

Conclusion

We sincerely thank the Editor and Reviewer for their constructive feedback, which has greatly improved the quality and clarity of our manuscript. We hope that the revised version meets the journal’s expectations and would be pleased to provide any further revisions if required.

\
